# Death associated protein kinase 2 suppresses T-B interactions and GC formation

**DOI:** 10.1016/j.molimm.2020.10.018

**Published:** 2020-12

**Authors:** Xingya Ni, Yifeng Wang, Pei Wang, Coco Chu, Heping Xu, Jinzhi Hu, Jiahui Sun, Hai Qi

**Affiliations:** a Tsinghua-Peking Center for Life Sciences, China; b Laboratory of Dynamic Immunobiology, Institute for Immunology, China; c Department of Basic Medical Sciences, School of Medicine, China, Tsinghua University, Beijing 100084, China; d Beijing Key Laboratory for Immunological Research on Chronic Diseases, Tsinghua University, Beijing 100084, China; e Beijing Frontier Research Center for Biological Structure, Tsinghua University, Beijing 100084, China

**Keywords:** Dapk2, Germinal center, T-B cell interaction, Raptor, mTORC1

## Abstract

Germinal center (GC) formation is a critical step during T-dependent humoral immune responses. We report Death Associated Protein Kinase 2, a serine/threonine kinase, is rapidly induced in T cells following activation and plays an inhibitory role in T cell-mediated help for the GC formation. Specifically, T cells deficient in Dapk2 have an increased ability to physically conjugate with antigen-presenting B cells and to promote GC formation. However, Dapk2 does not regulate T cell receptor signaling strength and does not influence cytokine-driven T-cell subset polarization. Instead, Dapk2 dampens mTORC1 activities by associating with Raptor. Silencing of Raptor rescues defects observed with the Dapk2 insufficiency. Our study thus identifies Dapk2 as a new kinase likely involved in negative regulation of contact-dependent help delivery to B cells and GC formation.

## Highlights

• The kinase Dapk2 is quickly induced in activated T cells.
• Dapk2 deficient T cells promote GC responses by increasing cognate T-B interactions.
• mTORC1 pathway mediates Dapk2 function in T cell.

## 1 Introduction

GC is the site for B-cell somatic hypermutation, affinity-based selection and the generation of long-lived humoral immune memory (Victora and Nussenzweig, 2012). GC development critically depends on contact-dependent T cell help (Biram et al., 2019; De Silva and Klein, 2015; Nutt and Tarlinton, 2011; Qi, 2016), particularly in the form of CD40 Ligand (CD40L), which promotes B-cell expansion by activating the CD40 receptor. Efficient delivery of CD40L and other contact-dependent T-cell help relies on physical interactions between antigen-specific T and B cells. Antigen-specific T-B interactions initially take place at the T-B border (Garside et al., 1998) and assume a form of long-lasting cell-cell conjugates *in vivo* (Okada et al., 2005). Following this early phase of cognate T-B interactions, T cells migrate into the follicle in conjunction with activated B cells and become follicular helper T (Tfh) cells that highly express chemokine receptor CXCR5, co-signaling molecule ICOS and PD-1, and the transcription factor Bcl6 (Crotty, 2011; Pratama and Vinuesa, 2014; Wan et al., 2019). While the precise relationship between so-defined Tfh cells and other helper subsets defined by cytokine profiles is still debated, it has become clear that Tfh cells can produce a variety of effector cytokines including IL-4, IL-9, IL-10, IL-13, IL-17, IL-21, and IFN-γ (Chtanova et al., 2004; Gowthaman et al., 2019; Linterman et al., 2011; Nakayamada et al., 2011; Reinhardt et al., 2009; Smith et al., 2016; Wang et al., 2017). Each of these cytokines has been shown to play a specific role in regulating certain aspects of the GC response.

Stable physical interactions between antigen-specific T and B cells are of central importance to T-dependent B-cell clonal expansion and GC formation. The stability of T-B interactions is maintained by T cell receptor (TCR) signaling, which enhances integrin-mediated intercellular adhesion (Billadeau et al., 2007; Burkhardt et al., 2008; Negulescu et al., 1996). As such, molecules and pathways that promote TCR signaling would enhance the stability of T-B interactions. For example, the SAP adaptor protein is crucial for stable T-B interactions because it protects proximal TCR signaling from being inhibited by SHP-1-associated Ly108 molecules (Chu et al., 2014; Kageyama et al., 2012; Qi et al., 2008). Besides inside-out signaling to integrins following TCR activation, other molecules and signaling pathways that modulate T-B cell interactions are less well characterized.

Mammalian target of rapamycin (mTOR) is an integrator of environmental cues for metabolic regulation of cellular physiology (Wullschleger et al., 2006). TCR stimulation, particularly in combination with costimulatory signals, markedly enhance mTOR activities mediated by the two mTOR complexes, mTORC1 and mTORC2, that are supported by scaffold protein Raptor and Rictor respectively (Chi, 2012). Consequently, mTOR functions are deeply involved in almost all aspects of T cell biology, including subset development and regulation of T-dependent help for the B-cell response (Pollizzi and Powell, 2015; Zeng and Chi, 2013). It is not known whether mTOR is directly involved in regulating antigen-specific T-B interactions.

Death associated protein kinase 2 (Dapk2) is a calcium/calmodulin regulated Ser/Thr kinase that belongs to the DAPK family (Kawai et al., 1999). Like other members of this family, Dapk2 has been reported to modulate cell death in response to different stimuli (Bialik and Kimchi, 2006). For example, in HEK293 and 3T3 cells, Dapk2 overexpression leads to membrane blebbing, a characteristic feature of apoptosis. Dapk2 has also been found to regulate mTOR activities and autophagic cell death (Ber et al., 2015). In innate immune cells, Dapk2 is implicated in regulation of chemotaxis and may promote cell recruitment during inflammation (Geering et al., 2014). Very little is known about whether and how DAPK2 functions in adaptive immunity. In this study, we identify Dapk2 as a novel modulator of the T-dependent B cell response. We find that Dapk2 is highly expressed in activated T cells, inhibits mTORC1 activities in T cells, suppresses stable T-B interactions *in vitro* and negatively regulates T-dependent GC formation *in vivo*.

## 2 Materials and methods

### 2.1 Mice

C57BL/6 (Jax 664), *Tcrb*^−/−^*Tcrd*^−/−^ (Jax 002122), dsRed-expressing (Jax 6051), OVA_323–339_-specific T cell receptor transgenic OT-2 mice (Jax 4194), and HEL-specific Ig-transgenic MD4 (Jax 2595) mice were from the Jackson Laboratory. For germline deletion of *Dapk2*, the CRISPR/Cas9 technique was used. Briefly, guide RNAs targeting two different sites within the exon 3 of *Dapk2* were injected into fertilized oocytes from C57BL/6 mice together with the Cas9 mRNA. F0 animals were then screened for deletion of the targeted genomic region by both PCR and Sanger sequencing. A founder mouse carrying an 85 bp deletion within the open reading frame of *Dapk2*, which results in a premature stop codon, was chosen and backcrossed to the parental C57BL/6 strain for at least 3 generations before the homozygous knockout animals were generated. The *Dapk2* knockout mice were genotyped using the following primers: 5′- CCCTTAGGAGTAGAAGGGAATTCAG-3′ (sense); 5′- AGACGTCGTGCAGCGTGATGATGTT-3′ (antisense). *Dapk2* KO OT-2 mice were further generated by interbreeding relevant strains. All mice were maintained under specific-pathogen free conditions. All experiments involving mice were approved by the Institute Animal Care and Use Committee of the Tsinghua University in accordance with governmental guidelines for animal welfare.

### 2.2 shRNA Knockdown

shRNA targeting *Dapk2*, *Rptor* and *Tsc1* were cloned into the MSCV-LMP vector (gift from Dr. Yun-Cai Liu), a mir-30 based retroviral vector, which allows co-expression of shRNAs and the mAmetrine fluorescent protein. The knockdown efficiency of each shRNA was confirmed by transduction of primary CD4 T cells *in vitro* followed by quantitative PCR measurements of target gene mRNA levels. The sequences of the shRNAs used in the study are as follows: *Dapk2*- TGGCCAGTTTGCCATCGTGAAG; *Rptor*- GCCCGAGTCTGTGAATGTAATG; *Tsc1*- AACTCATGATGTTGTAATAGAG.

### 2.3 Quantitative PCR analysis

The mRNAs were extracted using the RNeasy Mini Kit (QIAGEN), and reverse-transcribed into cDNAs using the First Strand cDNA Synthesis Kit (Thermo Scientific). Quantitative PCR was performed using the following primers: *Hprt* (sense: 5′- CTTGCTGGTGAAAAGGACCTCTC-3′, antisense: 5′- GAAGTACTCATTATAGTCAAGGGCA-3′); *Dapk2* (sense: 5′- CGGAAACGGCTTACCAT-3′, antisense: 5′- CGCCTGCGGACATACTG-3′); *Rptor* (sense: 5′- GTCGCCTCTTATGGGACTCG-3′, antisense: 5′- ATTCAGGCACAGGACCAAGG-3′); *Tsc1* (sense: 5′- GGGAGCTGTTCCGTAATAAGAG-3′, antisense: 5′- GCATCTAGACTCAGGGAAGTC-3′).

### 2.4 Cell culture and retroviral transduction

CD4^+^ T cells and CD19^+^ B cells were isolated from spleen using the CD4 (L3T4) MicroBeads and the CD19 MicroBeads (Miltenyi Biotec), respectively, according to the manufacturer’s protocol. For *in vitro* T cell activation, CD4 T cells were stimulated with 8 μg/mL plate-bound anti-CD3 and anti-CD28 antibodies (BioXCell), and cultured in complete RPMI medium containing 10 ng/mL IL-2 (PeproTech) for 5 days before being used in subsequent experiments. To produce retrovirus, the gene of interests were first cloned into MSCV or PIB-based bicistronic vectors, which allow expression of the green or mAmetrine fluorescent proteins along with the target genes. Retrovirus was produced through transfection of retroviral vectors into the platinum-E cell lines (Cell Biolabs). Subsequently, primary T cells activated for 24 h were infected by centrifugation for 2 h at 32℃, 1500 *g* with viral supernatant supplemented with 10 ng/mL IL-2 and 1 μg/mL polybrene (sigma).

### 2.5 Adoptive transfer and immunization

To study GC responses with OT-2 T cells as helpers, 5 × 10^5^ OT-2 T cells and 5 × 10^5^ MD4 B cells were intravenously transferred into *Tcrb*^−/−^*Tcrd*^−/−^ mice, and the recipients were subcutaneously immunized one day later with 130 μg HEL-OVA mixed with 1 μg lipopolysaccharide (LPS) (Sigma) in alum (Thermo Scientific). When T cell transduction was required, 1 × 10^6^ infected OT-2 T cells were sorted 5 days after activation, and transferred to C57BL/6 mice together with 5 × 10^5^ MD4 B cells. The recipients were immunized 3 days later as mentioned above.

### 2.6 T-cell stimulation

For each condition, 2 × 10^6^ *in vitro* activated T cells were incubated on ice with 20 μg/mL biotinylated anti-CD3 antibodies (145-2C11, eBioscience) in serum-free RPMI medium for 30 min, washed twice, and pre-warmed at 37 °C for 5 min. To crosslink TCRs, 10 μg of pre-warmed streptavidin (Roche) were added to T cells for the indicated length of time. The cells were subsequently lysed by the addition of equal volume of ice-cold lysis buffer (100 mM Tris (pH 8.0), 2% Triton X-100, 4 mM EDTA) supplemented with protease (Sigma) and phosphatase inhibitors (Thermo Scientific).

### 2.7 Calcium influx assay

*In vitro* activated T cells were collected and stained with 2 mM Indo-1 (Invitrogen) in serum-free RPMI medium at 37 °C for 30 min, washed twice, and incubated in ice-cold RPMI containing 1% serum with 5 μg/mL biotinylated anti-CD3 antibodies for 30 min. The cells were then washed twice and warmed to 37 °C for 5 min. Baseline fluorescent signals of Indo-1 were analyzed by flow cytometry for 1 min before the addition of streptavidin (40 μg/mL) for TCR crosslinking, and data acquisition resumes for another 4 min for the recording of calcium influx.

### 2.8 T-B conjugate assay

B cells were activated by 1 mg/mL LPS. Cells were collected after 2 days and, for most experiments, stained at 37 °C with 50 mM CMF_2_HC (Invitrogen) in PBS for 30 min, washed once, and then pulsed with OVA_323_- peptide at 37 °C for 30 min. *In vitro* activated OT-2 T cells were stained at 37 °C with 5 mM TAMRA (Invitrogen) in PBS for 30 min. In some experiments when non-transduced T cells were analyzed, for convenience, T cells and peptide-pulsed B cells were stained on ice for 30 min with fluorescent anti-CD4 and anti-CD19 antibodies instead of with fluorescent dyes. For conjugation, 2.5 × 10^5^ T cells were mixed with 5 × 10^5^ antigen pulsed B cells in a 5 mL round bottom test tube (BD Biosciences), centrifuged at 300 *g*, and incubated at 37 °C for 30 min. T-B conjugates were analyzed by flow cytometry immediately after resuspension of the cell pellet by vortexing for 30 s.

### 2.9 T-cell polarization assay

Naïve T cells (CD4^+^CD25–CD44^lo^CD62L^hi^) were sorted and activated by 5 μg/mL plate-bound anti-CD3 and anti-CD28 antibodies in complete RPMI medium with the indicated cytokines and antibodies (Th0: 10 μg/mL of anti-IL-12, anti-IFNγ and anti-IL-4; Th1: 10 ng/mL IL-12 and IFNγ, 10 μg/mL anti-IL-4; Th2: 100 ng/mL IL4, 10 μg/mL anti-IL-12 and anti-IFNγ; Th17: 1 ng/mL TGFβ and 20 ng/mL IL-6; Treg: 2 ng/mL TGFβ).

### 2.10 Immunoprecipitation and immunoblotting

Cells were washed with PBS and lysed on ice for 15 min with lysis buffer (40 mM HEPES, 2 mM EDTA, 0.3 % CHAPS, protease (Sigma) and phosphatase inhibitor mixtures (Thermo Scientific)). Cell lysates were centrifuged at 4 °C for 10 min at 21,000 g, and the supernatants were incubated with anti-FLAG M2 beads (Sigma) overnight at 4 °C. The beads were then washed five times with lysis buffer and the immune-precipitated (IP) protein was collected through boiling of the beads with SDS containing sampling buffer for 10 min. For western blot analysis, cell lysates or the IP proteins were separated by SDS-PAGE and transferred to PVDF membranes. The membranes were blocked with 5% milk in TBS buffer containing 0.1 % Tween-20 and subsequently incubated with different antibodies (anti–Dapk2 (Abcam); anti-Flag, anti-HA (Sigma); anti-pPLCγ1 (Y783) (Thermo Scientific); anti-pErk1/2 (T202/Y204), anti-pS6K1 (T389), anti-pAkt (T308), anti-PLCγ1, anti-Erk1/2, anti-S6K1, anti-Akt, anti-Raptor and anti-Actin (Cell Signaling Technology)). The membranes were then stained with matching HRP-conjugated secondary antibodies to allow visualization of target proteins (Jackson Immunoresearch Laboratories). Densitometry for protein bands was analyzed with the AlphaView software (ProteinSimple).

### 2.11 Flow cytometry

Cells from draining lymph nodes were first blocked with 20 μg/mL 2.4G2 antibodies (BioXcell) then stained with indicated antibodies in MACS buffer (PBS supplemented with 5 mM EDTA and 1% FBS). Staining reagents are as follows: PE-Cy7 anti-CD4, efluor450 anti-CD4, APC-Cy7 anti-B220, APC-Cy7 anti-CD19, PE-Cy7 anti-CD95, and PE anti-IgMa (BD Biosciences); efluor450 anti-GL7, streptavidin-PE, streptavidin-APC (eBioscience); FITC anti-CD4, PE-Cy7 anti-PD1 (Biolegend); biotinylated anti-CXCR5 and PE anti-CXCR5 (Miltenyi). Isotype-matched control antibodies were also used when necessary. Cells were first incubated with primary antibodies on ice for 30−90 min, washed with MACS buffer, and incubated with secondary antibodies for an additional 30 min. Data were collected on either LSR II or Aria III cytometer (BD Biosciences) and analyzed with the FlowJo software (TreeStar). Dead cells and cell aggregates were excluded from the analyses based on characteristics of forward- and side-scattering and staining of 7-amino-actinomycin D (7-AAD) (Biotium).

### 2.12 Statistical analyses

For all animal experiments, sex- and age-matched mice were randomly chosen for different treatment groups. Each group was typically consisted of at least 3 animals, and experiments were repeated 3–4 times for each setup. Statistical tests were performed with the Prism software (GraphPad). Unless specifically indicated, two-tailed t tests were performed to compare endpoint means of different groups. Error bars denotes the s.e.m..

## 3 Results

### 3.1 Dapk2 negatively regulates helper T cell functions underlying GC formation

**Fig. 1:**
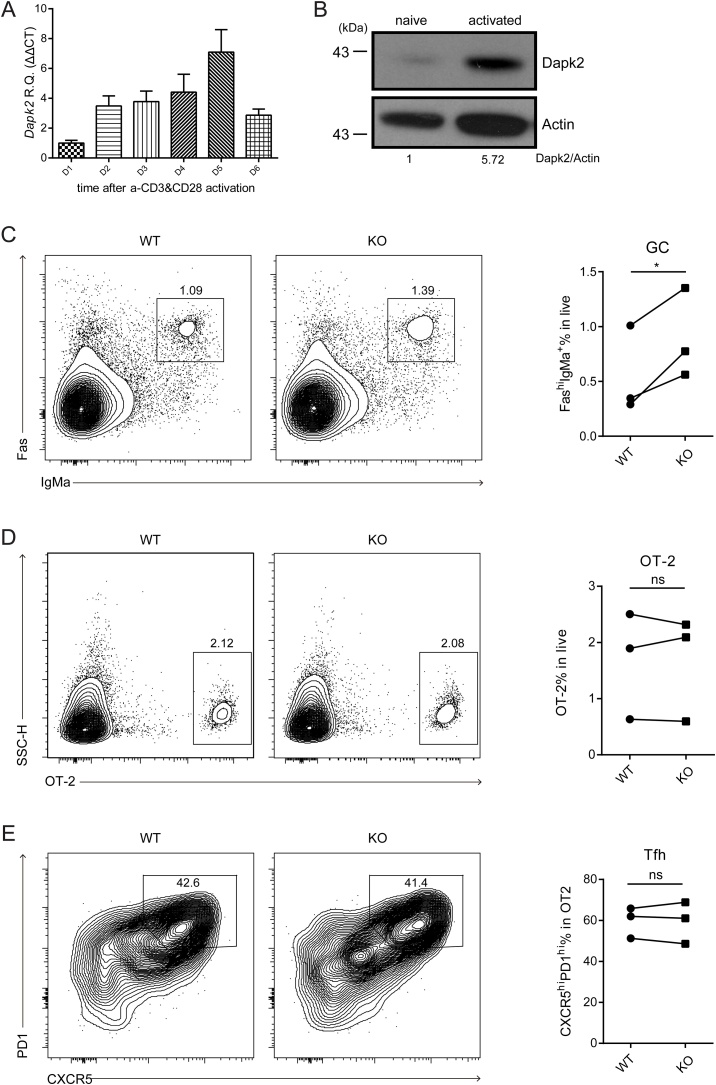
Dapk2 deficiency in T cells leads to increased GC responses. (A) Expression of *Dapk2* mRNA increases upon TCR activation. The mRNA level of *Dapk2* is analyzed by qPCR and normalized to the level of Hprt, before and after *in vitro* activation of T cells through crosslinking of CD3 and CD28. Data are mean ± s.e.m. and representative of two independent experiments. (B) Western blot analysis of Dapk2 protein levels in naïve and *in vitro* activated CD4^+^ T cells. Data are representative of two independent experiments. (C—E) Adoptive transfer of dsRed OT-2 T cells from WT or *Dapk2* knockout mice together MD4 B cells into *Tcrb*^−/−^*Tcrd*^−/−^ recipients. Frequencies of transferred GC B, OT-2 T and Tfh cells in the recipient mice are shown as flow cytometry contour plots (Left) and statistical quantifications (Right). Each symbol represents the mean of one experiment, with at least 3 mice in each group per experiment. Symbols connected are from the same experiment. NS, not significant. *P < 0.05 (paired *t*-test).

To investigate possible functions of Dapk2 in T cells, we first examined the expression pattern of this kinase in CD4^+^ T cells. Upon *in vitro* activation of naïve CD4^+^ T cells with anti-CD3 and anti-CD28, Dapk2 expression increased by several folds and peaked at day 5 (Fig. 1A, 1B). A similar Dapk2 expression pattern was also found in CD8^+^ T cells (Fig. S1A). Importantly, Dapk2 is upregulated in both antigen-experienced CD4 and CD8 T cells *in vivo*, as demonstrated in a model of LCMV infection (Figs. S1B-C). Therefore, Dapk2 is rapidly induced and progressively upregulated following T cell activation.

We next generated Dapk2 knockout (KO) mice using the CRISPER/Cas9 technology (Figs. S2A-B). Dapk2 KO mice were born with an expected Mendelian frequency and showed grossly normal development of T cells in the thymus (Fig. S2C). Given the pattern of upregulation in activated T cells (Fig. S1B), we first tested the potential role of this kinase in development of different helper T cell subsets *in vitro*. As shown in Fig. S3, T cell polarization toward Th1, Th2 and Th17 subsets under cytokine-polarizing condition was not affected by the Dapk2 deficiency. It is difficult to generate *bona fide* Tfh cells *in vitro*. To examine potential influence of Dapk2 on Tfh development and function, we transferred into CD45.1 B6 mice Dapk2-sufficient or -deficient OT-2 T cells, which recognize I-A^b^-complexed OVA_323-336_ peptide epitope, together with hen egg lysozyme (HEL)-specific MD4 B cells. Five days after immunization with HEL-OVA conjugate antigen, no significant difference in Tfh development by OT-2 T cells or GC formation by MD4 B cells was observed (Fig. S4). Interestingly, however, when *Tcrb*^−/−^*Tcrd*^−/−^ mice that lack endogenous T cells were used as recipients, MD4 GC formation was enhanced in those receiving Dapk2 KO OT-2 helper T cells than those receiving WT T cells (Fig. 1C, shown as Fas^hi^IgMa^+^; for their universal GL7^+^ state, see Fig. S5A), although similar frequencies of CXCR5^hi^PD-1^hi^ Tfh cells were still observed in the two groups (Figs. 1D-E). These data indicate Dapk2 does not significantly influence Tfh development but could negatively regulate how T cells help B cells to form GCs.

### 3.2 Dapk2 suppresses T-B interactions

**Fig. 2:**
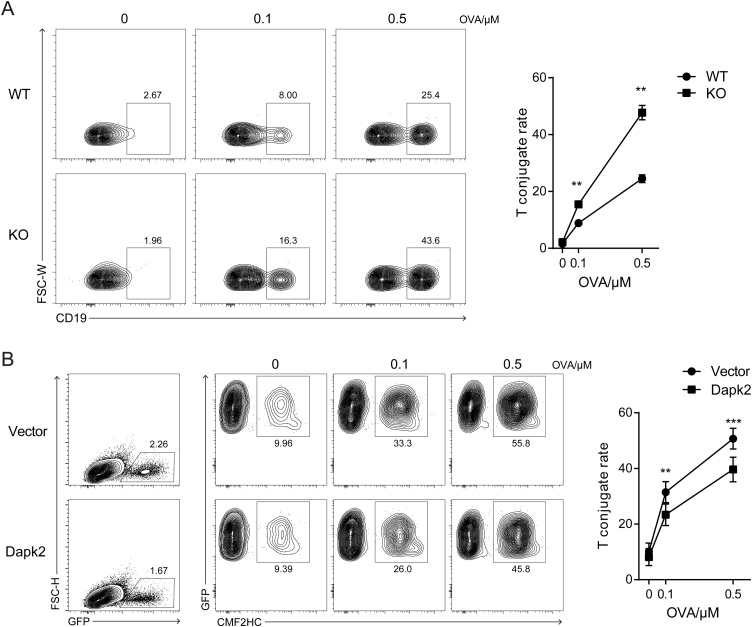
Dapk2 suppresses cognate T-B cell interactions. OT-2 T cells were stained with TAMRA or PE anti-CD4, and incubated with CMF2HC or ef450 anti-CD19 stained LPS-activated B cells pulsed with different concentration of OVA_323_-peptide. (A) Flow cytometry contour plots (left) and dot plots (Right) showing frequencies of WT or KO OT-2 T cells conjugated to B cells. Contour plots are gated on total CD4^+^ T cells. Data are mean ± s.e.m. from one of four independent experiments. **P < 0.01. (B) Flow cytometry contour plots (left) and dot plots (Right) showing conjugation frequencies of control or Dapk2 overexpressing OT-2 T cells to B cells. Contour plots are gated on total infected (GFP^+^) T cells. Data are mean ± s.e.m. and pooled from six independent experiments. **P < 0.01, ***P < 0.001.

Stable interactions between cognate T and B cells is crucial for delivery of T-cell help (Okada et al., 2005; Qi, 2016; Qi et al., 2008). To examine whether Dapk2 impinges on antigen-specific T-B interactions, we conducted *in vitro* conjugation assay (Chu et al., 2014; Qi et al., 2008) to compare Dapk2-sufficient and -deficient OT2 T cells. As shown in Fig. 2A, Dapk2 KO T cells formed more conjugates than did wildtype T cells with the B cells that were pulsed with OVA_323−336_ peptide, implying that Dapk2 negatively regulates T-B conjugation. To corroborate this interpretation, we further used shRNA to knock down Dapk2 in activated T cells and found that a significant reduction in Dapk2 expression would lead to enhanced T-B conjugation (Fig. S6). Furthermore, when T cells were retrovirally transduced to overexpress Dapk2, the rate of conjugation with B cells markedly decreased (Fig. 2B). These data indicate that Dapk2 suppresses T-cell interactions with cognate Ag-presenting B cells.

### 3.3 Dapk2 inhibits mTORC1 activity but not grossly TCR signaling in T cells

**Fig. 3:**
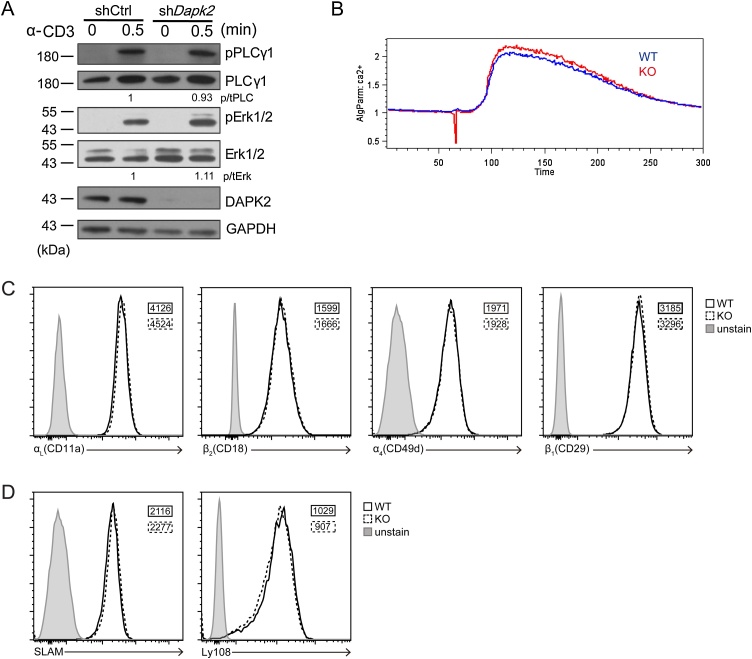
Proximal TCR signaling and integrin and SLAM family protein expression in Dapk2 KO T cells are intact. (A) PLCγ (Tyr783) and Erk1/2 (Thr202/Tyr204) phosphorylation in control or *Dapk2* shRNA transduced CD4^+^ T cells upon CD3 cross-linking. Data are representative of two independent experiments. (B-D) Calcium flux induced by CD3 crosslinking, expression of LFA-1 (αLβ2, CD11a/CD18), VLA-4 (α4β1, CD49d/CD29) (C), and Slam and Ly108 (D) are comparable in activated WT or KO CD4^+^ T cells. Data are representative of four (B) and two (C, D) independent experiments.

Antigen-specific T-B cell adhesion depends on antigen-triggered TCR signaling and is modulated by integrins and SLAM family proteins (Biram et al., 2019). To investigate how Dapk2 might increase antigen-specific T-B adhesion, we first explored whether Dapk2 alters TCR signaling. As shown in Fig. 3A, upon anti-CD3 stimulation, control and Dapk2-suppressed T cells exhibited similar PLC-γ1 and Erk phosphorylation. Furthermore, wildtype and Dapk2 KO T cells exhibited similar calcium fluxes in response to anti-CD3 stimulation (Fig. 3B). These data indicate Dapk2 does not suppress TCR signaling. Besides, expression of integrin LFA-1, VLA-4 and SLAM family protein Ly108 and Slam in T cells were not altered due to a Dapk2 deficiency (Figs. 3C-D).

**Fig. 4:**
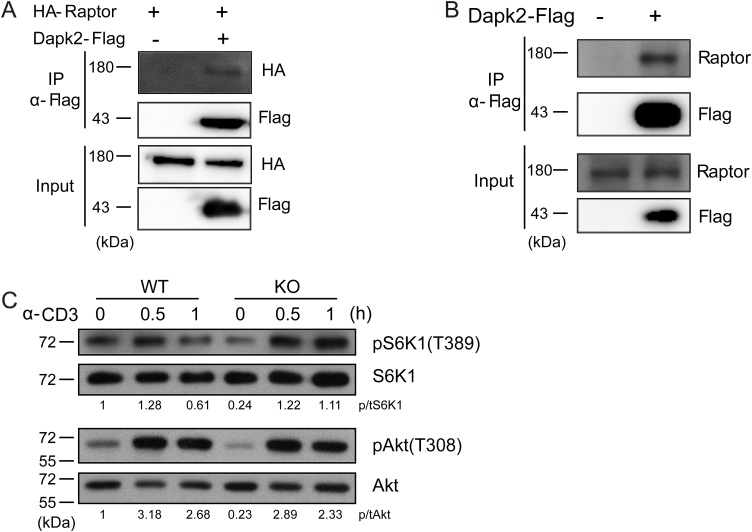
Dapk2 interacts with Raptor to dampen TCR-induced mTORC1 signaling. (A-B) Flag-tagged Dapk2 was immunoprecipitated from lysates of transduced HEK293 T cells that co-express HA-tagged Raptor (A) or transduced Jurkat T cells (B). The association between Dapk2 and Raptor were determined by western blot. (C) Time course analysis of S6K1 (T389) and Akt (T308) phosphorylation after CD3 cross-linking of WT or KO CD4^+^ T cells. Data are representative of two (A, B) or three (C) independent experiments.

We thus searched for other pathways that could be modulated by Dapk2 in T cells. Dapk2 has been shown in HEK293 cells to bind to and phosphorylate Raptor and to suppress mTORC1 activities (Ber et al., 2015). Suppression of mTORC1 activities could underlie the negative regulation Dapk2 exerts on T cells. To investigate this possibility, by immunoprecipitation we first reproduced previous observations that in HEK293 cells Dapk2 is associated with Raptor (Fig. 4A). We then examined in the Jurkat T cell line and found similar Dapk2-Raptor association (Fig. 4B). Functionally, upon anti-CD3 stimulation, Dapk2 KO T cells exhibited stronger phosphorylation of S6K1, indicative of increased mTORC1 activities (Fig. 4C). Notably, phosphorylation of Akt at the threonine residue 308, which is an upstream event leading to mTORC1 activation, was not significantly affected by the Dapk2 deficiency, in line with Raptor interactions as the main point of Dapk2-mediated regulation. A Dapk2 deficiency did not appreciably alter the cell size (Fig. S5B). These results indicate that Dapk2 dampens mTORC1 activities in T cells during T cell activation.

### 3.4 Raptor association underlies Dapk2 suppression of T-B interactions and T-cell help for GC formation

**Fig. 5:**
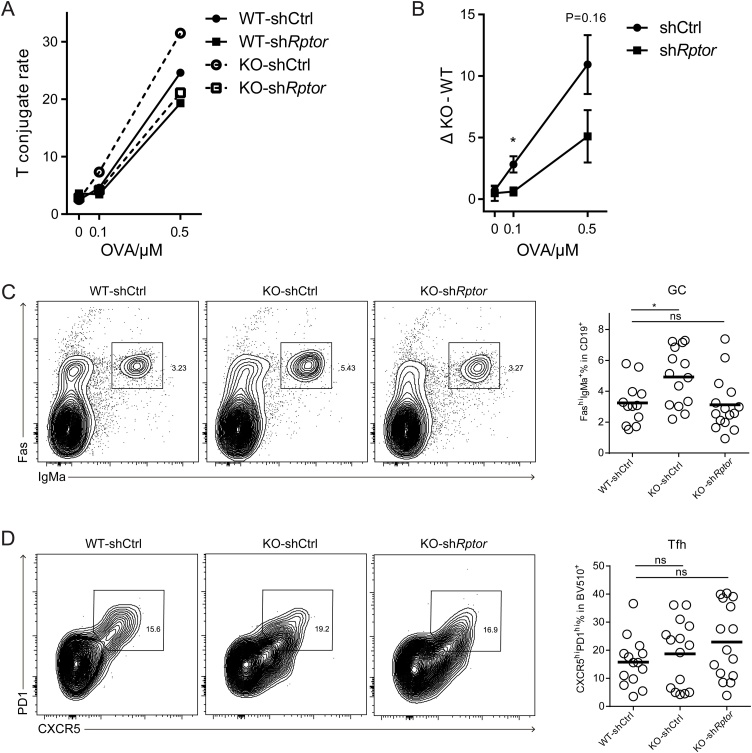
Dapk2 suppresses cognate T-B cell interactions and Tfh cell function through modulation of mTORC1 activities. (A) Frequencies of control or Raptor shRNA-transduced WT or Dapk2 KO OT-2 T cells in conjugation with LPS-activated, OVA_323_-pulsed B cells. Data are representative of three independent experiments. (B) Difference between conjugation frequencies of KO and WT OT-2 T cells transduced with either control or Raptor shRNAs. Data are mean ± s.e.m. and pooled from three independent experiments. (C, D) WT or KO OT-2 T cells were transduced with control or Raptor shRNAs and the transduced cells were isolated and transferred into B6 mice together with MD4 B cells. The recipients were immunized with HEL-OVA (s.c.) 3 days after the transfer and the analysis was done 5 days post immunization. The frequency of GC B cells (C) and Tfh cells (D) in the recipients are shown by flow cytometry contour plots (left) and statistical quantifications (right). Each symbol represents one mouse. Data are mean ± s.e.m. and pooled from four independent experiments. NS, not significant. *P < 0.05 (unpaired *t*-test).

To investigate whether Dapk2 suppresses T-B interactions by repressing mTORC1 activities, we transduced wildtype or Dapk2 KO OT-2 T cells with control (shCtrl) or Raptor-suppressing (sh*Rptor*) shRNA vector and tested their conjugation with B cells. As shown in Fig. 5A, when Dapk2 was absent, the T-B conjugate frequency was increased (comparing WT-shCtrl and KO-shCtrl), as expected according to data presented in Fig. 2; however, this increase was diminished when Raptor was knocked down (comparing WT-sh*Rptor* and KO-sh*Rptor*). By calculating the conjugate frequency differentials between WT and KO from 3 independent experiments, we found that the inhibitory effect of Dapk2 on T-B conjugation was largely abrogated when Raptor was knocked down (Fig. 5B). These data support the conclusion that Dapk2 suppresses T-B conjugation by suppressing Raptor-promoted mTORC1 activities. Consistent with this conclusion, shRNA-mediated suppression of TSC1, the well-established mTORC1 repressor (Hay and Sonenberg, 2004), led to increased T-B cell conjugation (Fig. S7).

To test whether Dapk2-mediated inhibition of GC formation depends on Raptor *in vivo*, we conducted transfer experiments using Dapk2 KO OT-2 T cell transduced with control or Raptor-repressing shRNA together with MD4 B cells. As shown in Fig. 5C-D, whereas the frequency of CXCR5^hi^PD-1^hi^ Tfh cells remained comparable across all groups, GC formation was enhanced in the presence of Dapk2 KO OT-2 T cells, but the increase was abrogated when Raptor was knocked down. Together, our data suggest that in activated T cells Dapk2 inhibits Raptor and mTORC1 activities and thereby represses antigen-specific T—B adhesion and help delivery for GC formation.

## 4 Discussion

Our study suggests Dapk2 as an inhibitory regulator of T-cell help for GC formation. Cytokines produced by Th1, Th2, and Th17 cells have all been implicated in GC responses (Nakayamada et al., 2011; Reinhardt et al., 2009; Smith et al., 2016), although Dapk2 KO T cells show no appreciable defect in generating those cytokine-producing subsets *in vitro*. The inhibitory effect of Dapk2 on GC formation is likely due to its suppression of antigen-specific T-B adhesion. We further suggest that Dapk2 achieves this inhibition through modulation of mTORC1 activity.

The inhibitory effect of Dapk2 in the T-dependent B cell response is relatively subtle and appears to be context-dependent. In the adoptive transfer model, the inhibitory dapk2 effect is only observed when naïve OT-2 T cells are transferred into T cell-deficient but not wildtype recipients (Figs. 1C-E, Fig. S4). On the other hand, with regular wildtype B6 mice as recipients, the inhibitory dapk2 effect can be consistently revealed when shRNA-mediated knock-down is utilized, a procedure involving pre-activation *in vitro* (Figs. 5C-D). We speculate two possibilities. First, differences between models may be related to patterns of Dapk2 expression in T cells. Dapk2 expression is activation-dependent, and its level in OT-2 cells in the first setting might not have reached a level sufficient to suppress Raptor and mTORC1 when MD4 GC formation may already reach its peak. On the other hand, in the second setting of T cell-deficient environment, homeostatic proliferation in addition to HEL-OVA antigen stimulation may provide extra stimulation to increase Dapk2 expression. Second, because other members of the DAPK family are also expressed by B and T cells, and Drak2 has been found to be required for normal GC responses (Al-Qahtani et al., 2008), Dapk2 and Drak2 are possibly cross-regulated and effects of Dapk2 manipulation might be compensated by altered Drak2 activities. Future studies are required to determine Dapk2 expression levels at various conditions and quantitatively describe the level at which Dapk2 begins to exert its inhibitory effect on mTORC1 and T-B interactions and to test potential cross-regulation between Dapk2 and other DAPK family members. Besides, because Dapk2 upregulation in activated CD8 T cells is even more pronounced, its role in the context of CD8 memory and exhaustion remains to be explored.

Previous studies have suggested either an inhibitory or supportive role for mTORC1 activities in regulation of Tfh development (Ray et al., 2015; Zeng et al., 2016). Given the fundamental importance of the mTOR pathway for cellular physiology, a potential scenario reconciling the contradiction is that Tfh cells cannot efficiently develop when mTORC1 activities in T cells are below a basal level, as seen by Zeng et al. using Raptor conditional knockout (Zeng et al., 2016). On the other hand, beyond the basal level of mTORC1 activities, Tfh development appears inhibited by higher mTORC1 activities, particularly when induced by IL-2 stimulation or achieved in mTOR knock-down instead of knockout assays (Ray et al., 2015). In our study, we did not observe a difference in Tfh development, including Tfh percentage and cell sizes, following Dapk2 perturbation, which alters mTORC1 activities of T cells in response to TCR stimulation. This may be related to the fact that Dapk2 is tightly regulated by its autoinhibition at the steady state and requires binding of Ca^2+^/CaM for activation of its enzymatic activities (Shiloh et al., 2014). Conceivably, Dapk2 may exert its modulatory effects on mTORC1 activities in effector T cells only when such T cells receive antigen stimulation and generate strong TCR-dependent Ca^2+^ influx.

In terms of Tfh cell function, by shRNA-mediated Raptor silencing, we find mTORC1 activities can promote stable T-B interactions (Fig. S7C) and, consequently, help delivery to B cells and subsequent GC formation. Dapk2 regulates GC formation by modulating Raptor-controlled mTORC1 activities (Fig. 5C). It appears that only appropriate levels of mTORC1 activities in the helper T cell compartment are conducive to efficient T-cell help for the GC response. However, how Dapk2 attenuates T-B interactions by modulating mTORC1 activity remains to be elucidated. Surface integrins and SLAM family molecules are not likely the reason, because we did not observe Dapk2-deficient T cells exhibited any difference in upregulation of integrins and SLAM family molecules following activation *in vitro* (Fig. 3C-D). On the other hand, mTORC1 activities have been implicated in actin cytoskeletal reorganization during fibroblast migration, implying a potentially similar role for mTORC1 complex in T cells. Because mTORC2 regulates actin organization in a variety of cell types (Liu and Parent, 2011) and can be subjected to cross-inhibition by mTORC1 (Zoncu et al., 2011), we also speculate that Dapk2 might exert its effect on cytoskeleton dynamics and impact on T-B interactions via such a feedback through mTORC2-dependent pathway. Future work remains necessary to test these possibilities.

Despite the inhibitory effect of Dapk2 on T-B conjugation *in vitro*, by intravital microscopy we failed to observe obvious changes in duration of contact between antigen specific T and B cells *in vivo* (unpublished observations). We do not yet understand the precise reason for this discrepancy between *in vitro* conjugation assay and *in vivo* imaging observation, which in itself is a unique situation throughout our extensive experience in characterizing T-B interactions using various genetic perturbation models. We speculate there might be soluble factors that are differentially produced by WT and KO T cells and impact on their interactions with B cells *in vivo*, a hypothesis that we are pursuing currently.

## Funding

This work was supported in part by the National Key R&D Program of China (Ministry of Science and Technology, 2018YFE0200300), the 10.13039/501100001809Natural Science Foundation of China (8162002, 81761128019), and the Beijing Municipal Science and Technology Commission. This work was also funded in part by the Bill & Melinda Gates Foundation and the 10.13039/100000011Howard Hughes Medical Institute. The findings and conclusions within are those of the authors and do not necessarily reflect positions or policies of the Bill & Melinda Gates Foundation or the Howard Hughes Medical Institute.

## CRediT authorship contribution statement

**Xingya Ni:** Conceptualization, Formal analysis, Investigation, Writing - original draft, Writing - review & editing. **Yifeng Wang:** Conceptualization, Formal analysis, Investigation, Writing - original draft, Writing - review & editing. **Pei Wang:** Investigation, Resources. **Coco Chu:** Investigation, Methodology, Resources. **Heping Xu:** Methodology, Resources. **Jinzhi Hu:** Investigation, Resources. **Jiahui Sun:** Investigation, Resources. **Hai Qi:** Conceptualization, Funding acquisition, Methodology, Writing - original draft, Writing - review & editing.

## Declaration of Competing Interest

None.
