# Supplementary figures and images for: Death associated protein kinase 2 suppresses T-B interactions and GC formation
Source: Mol Immunol. 2020 Dec;128:249–57. doi: 10.1016/j.molimm.2020.10.018 (PMC7754787; doi:10.1016/j.molimm.2020.10.018)

# Fig S1

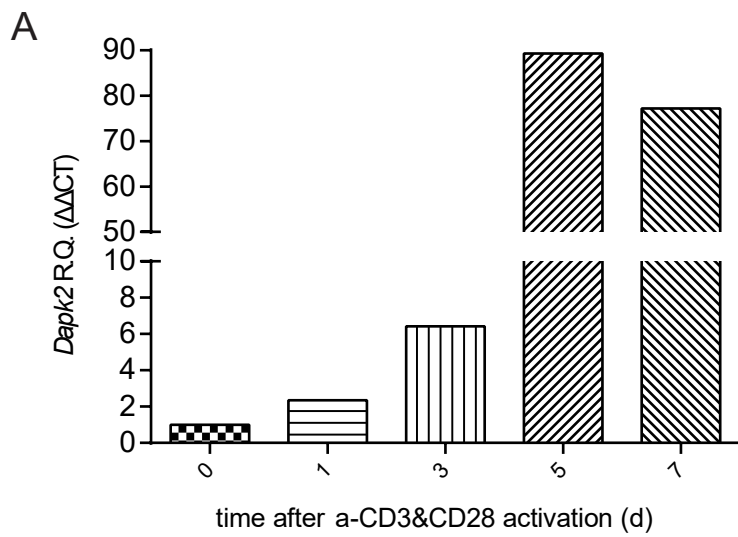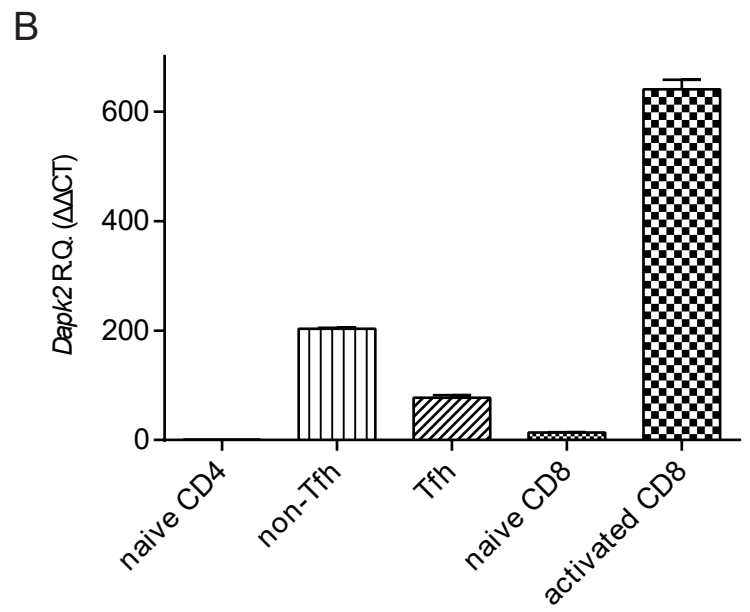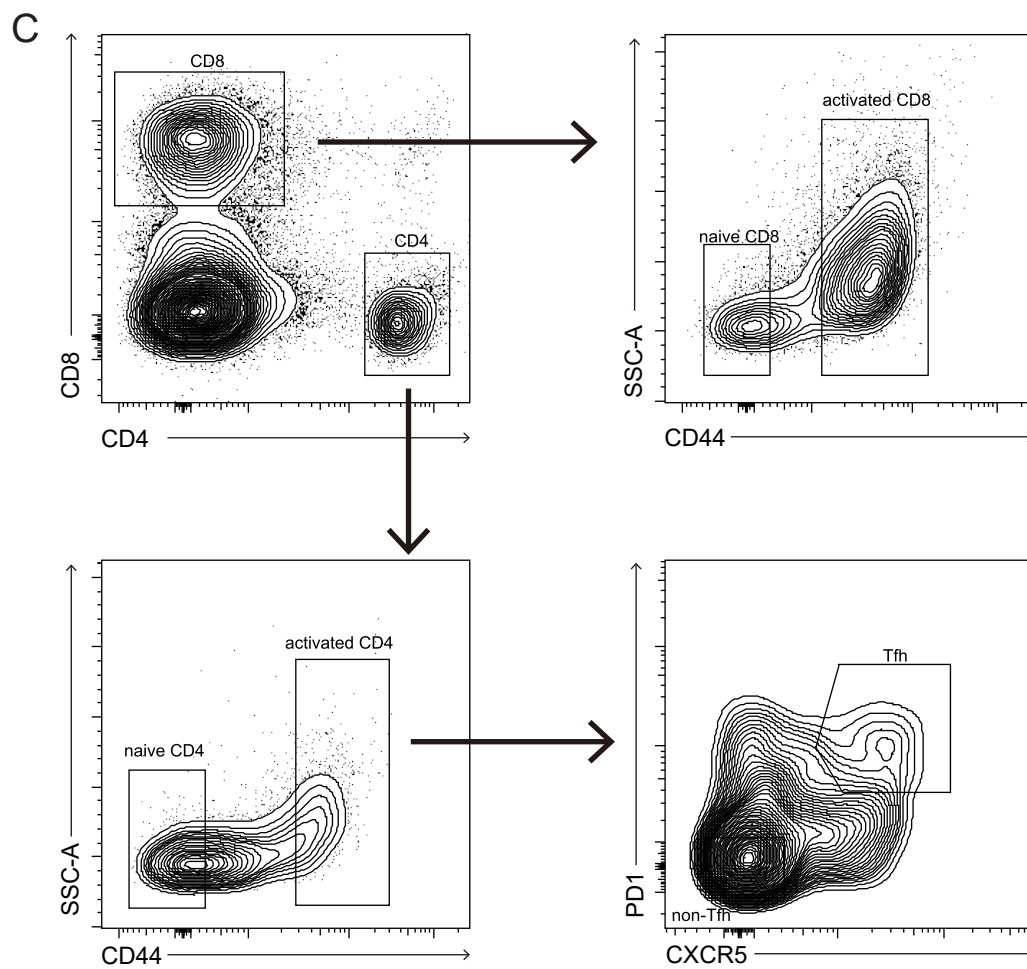

Supplement: Supplementary file 1 [file mmc1.pdf]

# Fig S2

A

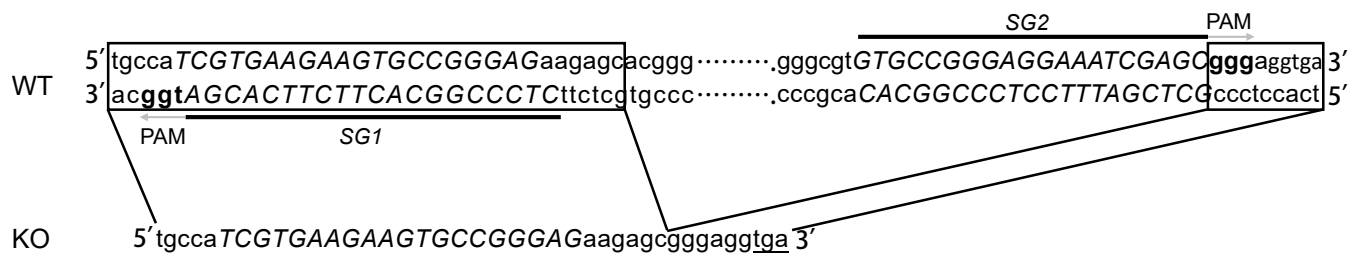

B

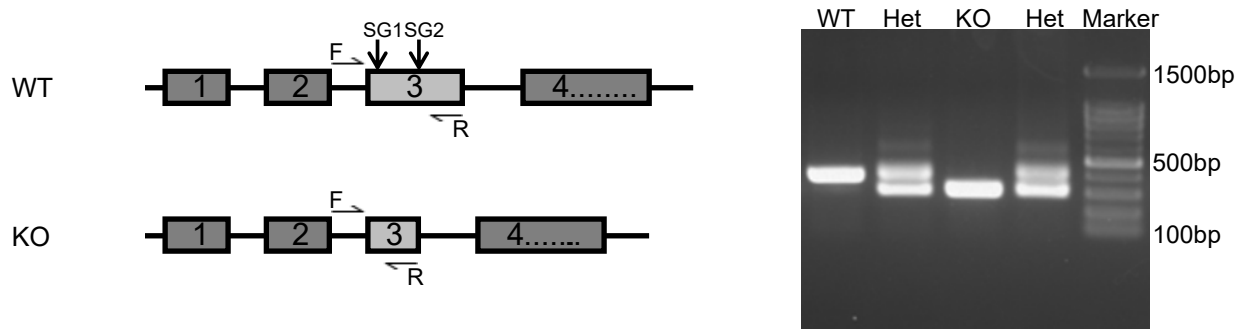

C

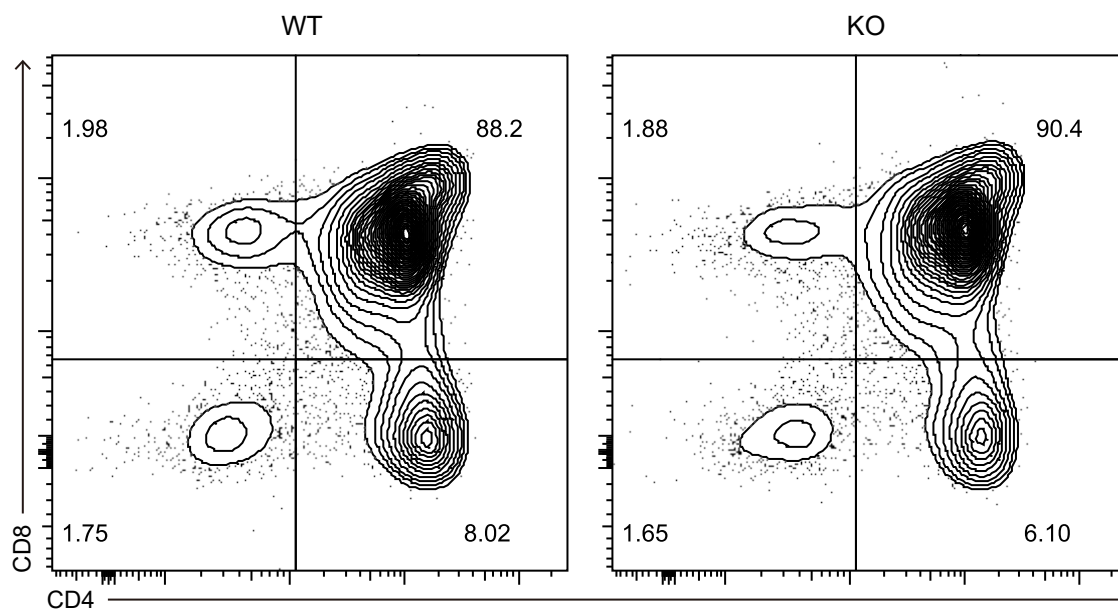

Supplement: Supplementary file 2 [file mmc2.pdf]

# Fig S3

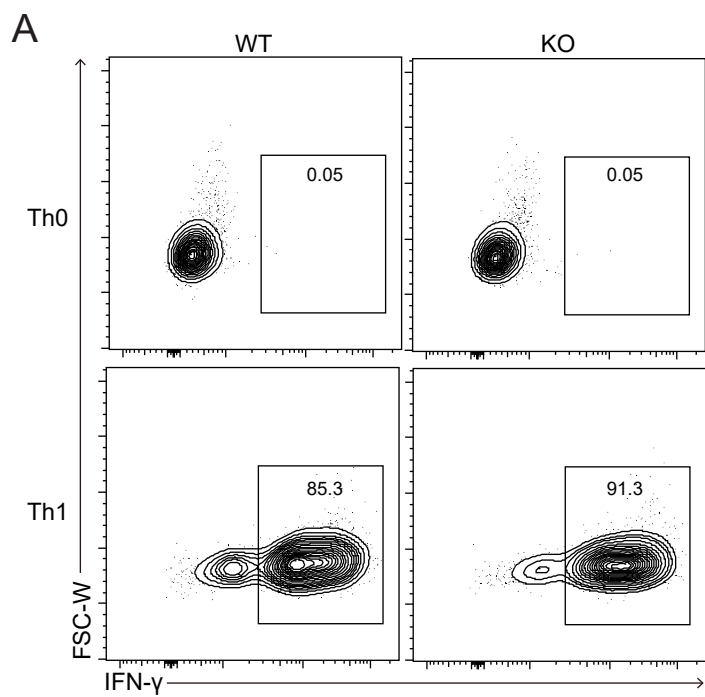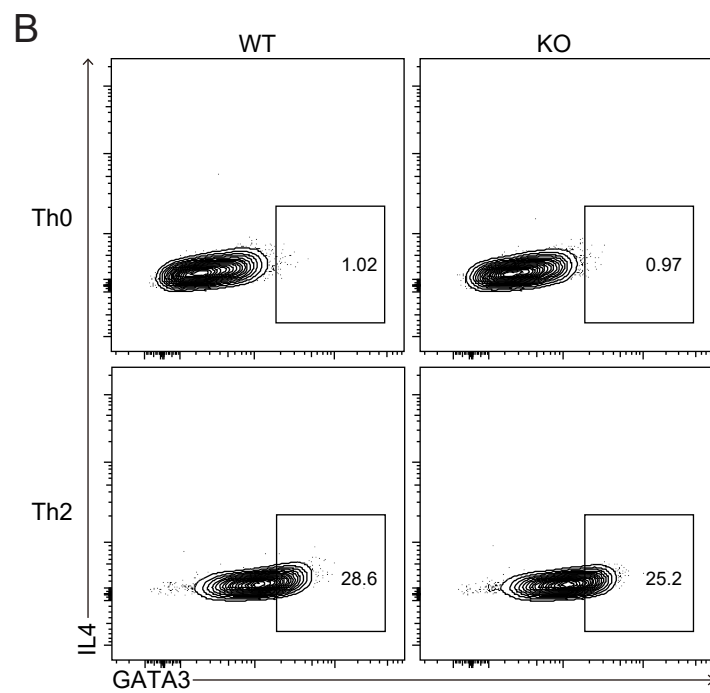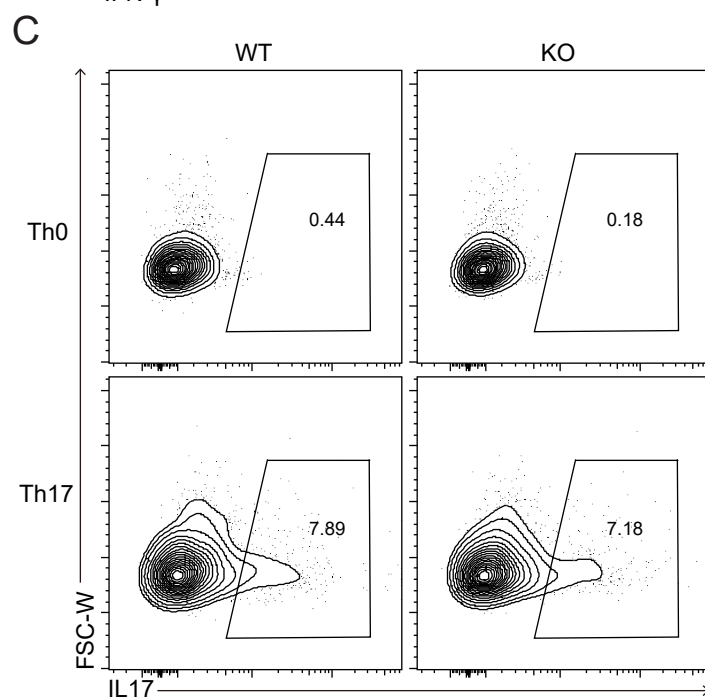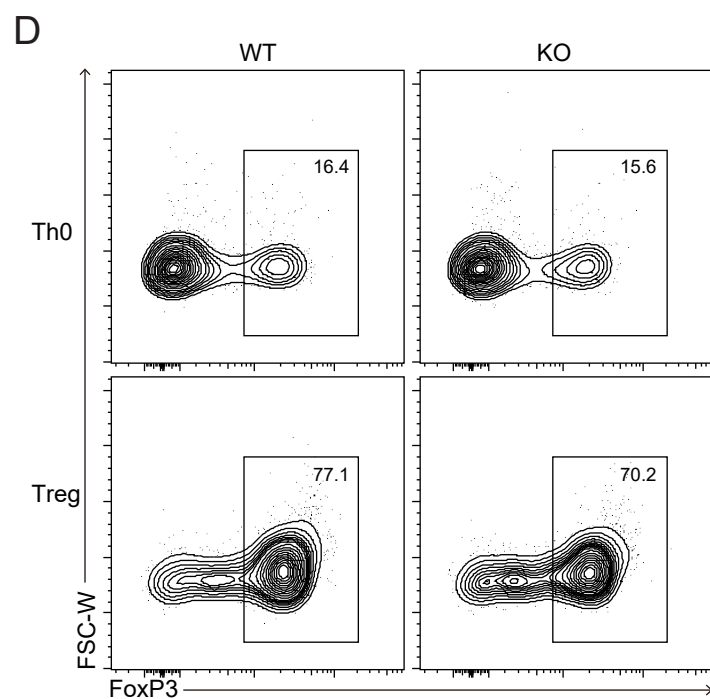

Supplement: Supplementary file 3 [file mmc3.pdf]

# Fig S4

A

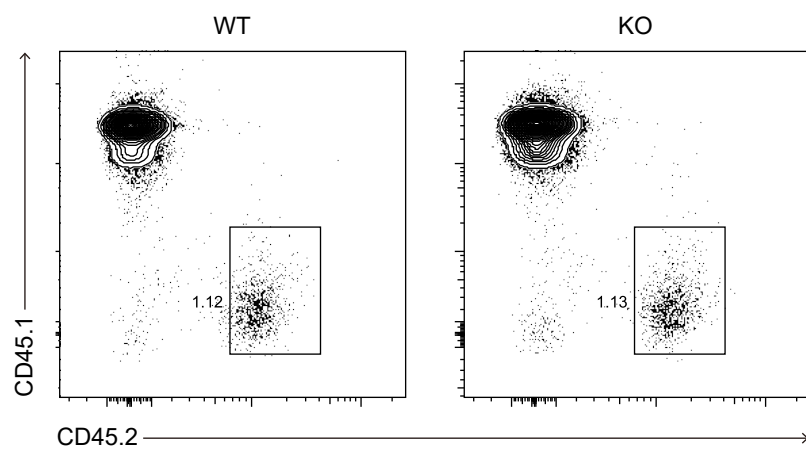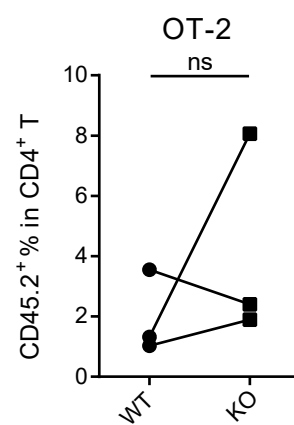

B

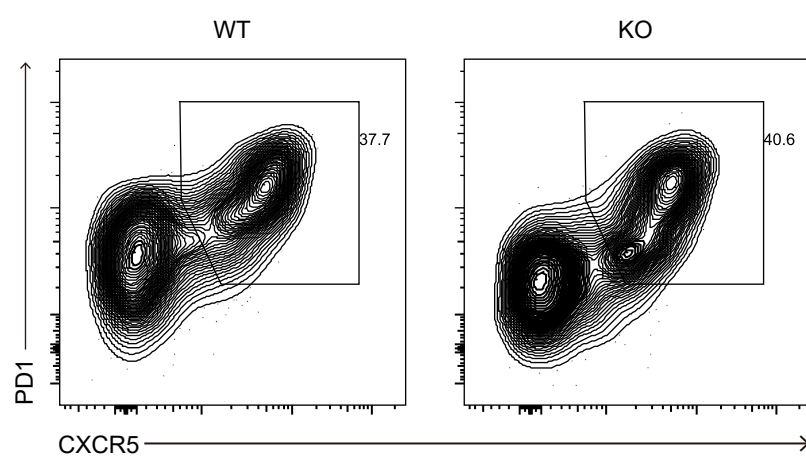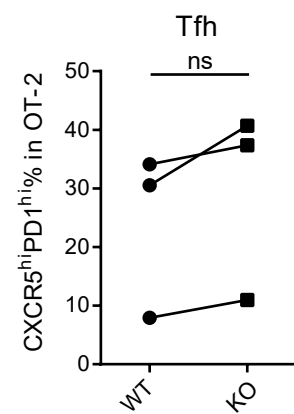

C

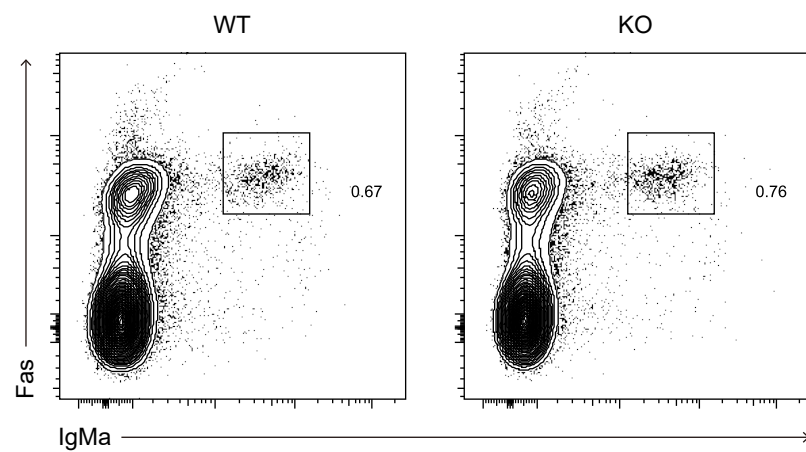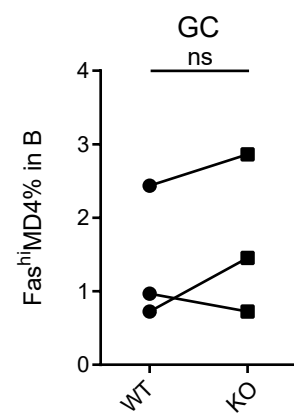

Supplement: Supplementary file 4 [file mmc4.pdf]

Fig S5

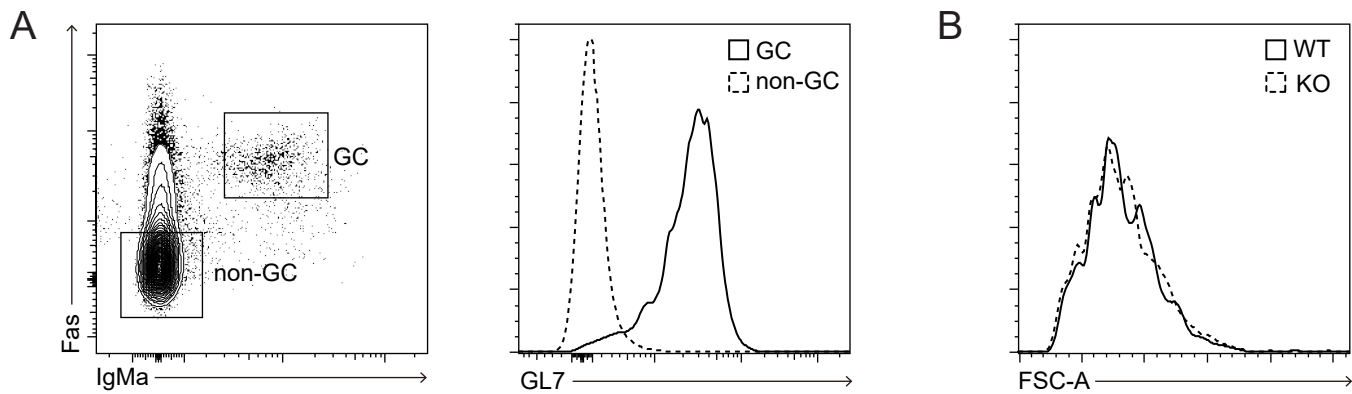

Supplement: Supplementary file 5 [file mmc5.pdf]

# Fig S6

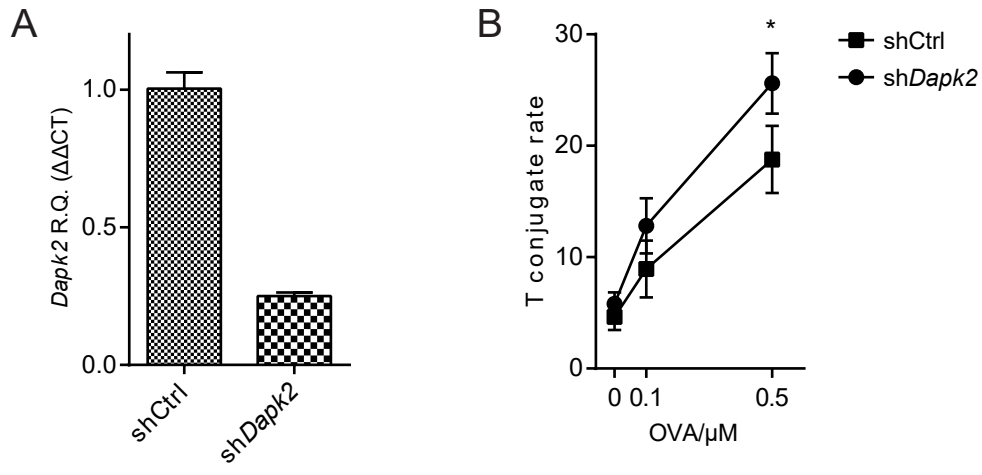

Supplement: Supplementary file 6 [file mmc6.pdf]

# Fig S7

A

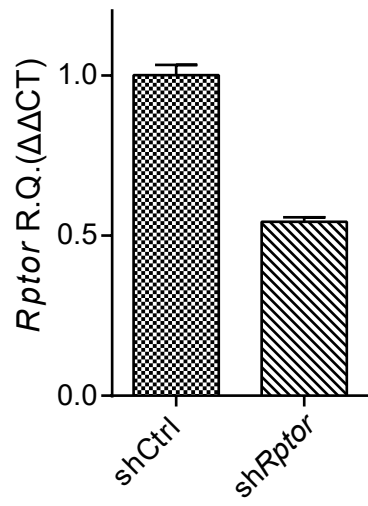

B

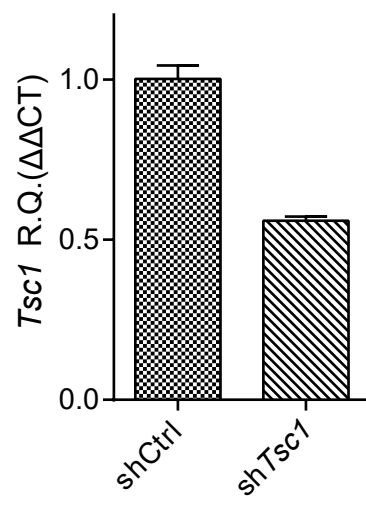

C

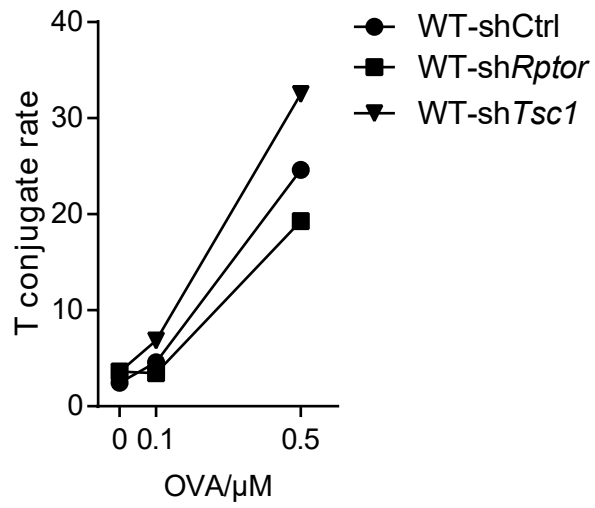

Supplement: Supplementary file 7 [file mmc7.pdf]
